# Exogenous ACC Deaminase Is Key to Improving the Performance of Pasture Legume-Rhizobial Symbioses in the Presence of a High Manganese Concentration

**DOI:** 10.3390/plants9121630

**Published:** 2020-11-24

**Authors:** Ana Paço, José Rodrigo da-Silva, Denise Pereira Torres, Bernard R. Glick, Clarisse Brígido

**Affiliations:** 1 MED—Mediterranean Institute for Agriculture, Environment and Development, Instituto de Investigação e Formação Avançada, Universidade de Évora, Pólo da Mitra, Ap. 94, 7006-554 Évora, Portugal; anaisapaco@gmail.com (A.P.); joserodrigope@gmail.com (J.R.d.-S.); deniseptorres@gmail.com (D.P.T.); 2 Department of Biology, University of Waterloo, Waterloo, ON N2L 3G1, Canada; glick@uwaterloo.ca

**Keywords:** abiotic stress, plant–bacteria interactions, plant growth promotion, endophytes, plant growth-promoting traits, nodulation, acidity

## Abstract

Manganese (Mn) toxicity is a very common soil stress around the world, which is responsible for low soil fertility. This manuscript evaluates the effect of the endophytic bacterium *Pseudomonas* sp. Q1 on different rhizobial-legume symbioses in the absence and presence of Mn toxicity. Three legume species, *Cicer arietinum* (chickpea), *Trifolium subterraneum* (subterranean clover), and *Medicago polymorpha* (burr medic) were used. To evaluate the role of 1-aminocyclopropane-1-carboxylate (ACC) deaminase produced by strain Q1 in these interactions, an ACC deaminase knockout mutant of this strain was constructed and used in those trials. The Q1 strain only promoted the symbiotic performance of *Rhizobium leguminosarum* bv. trifolii ATCC 14480^T^ and *Ensifer meliloti* ATCC 9930^T^, leading to an increase of the growth of their hosts in both conditions. Notably, the *acdS* gene disruption of strain Q1 abolished the beneficial effect of this bacterium as well as causing this mutant strain to act deleteriously in those specific symbioses. This study suggests that the addition of non-rhizobia with functional ACC deaminase may be a strategy to improve the pasture legume–rhizobial symbioses, particularly when the use of rhizobial strains alone does not yield the expected results due to their difficulty in competing with native strains or in adapting to inhibitory soil conditions.

## 1. Introduction

Acid soils represent ≈30% of the total area of the planet and 50% of the world’s arable land [1]. Soil acidity restricts agricultural production mainly due to nutrient deficiency and toxicity by metals such as manganese (Mn), iron (Fe), and aluminum (Al), with Al and Mn toxicities being the main factors limiting plant growth in acidic soils [2,3,4]. Therefore, Mn toxicity is a very common stress in arable lands around the world [5], and in particular, it is one of the most important limiting factors to plant productivity in the South of Portugal [6]. In this regard, the most common production system in Portugal, mainly in the Alentejo region in Southern Portugal, is the silvo-pastoral Montado ecosystem [7]. Animal production is an important component of the Montado system, but the natural pastures in these soils are unproductive, and the success of sown pastures, in addition to small grains, is greatly reduced due to very high soil acidity and Mn concentrations [8]. The recommended procedure for the recovery of these soils is the installation of permanent pastures and increasing soil fertility through the application of chemical phosphate fertilizers [9] or the correction of soil acidity by the addition of dolomitic limestone [10]. However, either of these approaches is expensive and not available to farmers in large areas of the world, and hence, it is important to develop less expensive solutions.

Based on the diverse expressions of Mn toxicity, it may be assumed that tolerance to Mn depends on the plant species and cultivar. Among different pasture legumes, white clover (*Trifolium repens* L.), alfalfa (*Medicago sativa* L.), and barrel medic (*M. truncatula* L.) are considered to be very sensitive to Mn toxicity [11], while subterranean clover (*T. subterraneum* L.) is considered to be relatively tolerant to soil acidity [12]. In addition, as a consequence of the negative effects of Mn on the growth and activity of the root nodules, the negative effects of Mn on plant growth are more evident on N_2_-fixing plants than on plants supplied with mineral N [13,14]. Thus, improving the symbiotic performance of rhizobia with legumes growing in high acidic and high Mn soils through sustainable agricultural practices is a great challenge. 

Along with rhizobia, legumes can also establish associations with non-rhizobial endophytic bacteria, which also contribute to increases in plant biomass and yield [15]. Furthermore, their application in agriculture may be a promising and low-cost alternative either to increasing the availability of nutrients for the improvement of pasture productivity or to protect plants against Mn toxicity. In fact, the use of consortia, including both rhizobial and non-rhizobial endophytic bacteria, has been shown to synergistically enhance the symbiotic performance of rhizobia and consequently increase host plant growth [16,17,18], including under metal toxicity [19,20]. Moreover, studies on plant growth-promoting bacteria showed that one of the central mechanisms in plant-microbe interactions is the ability of some bacteria to synthesize the enzyme 1-aminocyclopropane-1-carboxylate (ACC) deaminase [21,22,23]. This enzyme acts to cleave the immediate ethylene precursor, ACC, into ammonia and α-ketobutyrate under free-living [24] or symbiotic conditions [25,26]. The importance of ACC deaminase for the symbiotic rhizobial-legume association has been demonstrated in several studies [27,28], particularly under stressful conditions [29,30,31,32]. 

Herein, it was hypothesized that the inoculation of plants with a consortium of rhizobia and non-rhizobial bacteria where at least one bacterium in the consortium possess ACC deaminase activity could be advantageous to promote legume growth, particularly under Mn stress conditions. Therefore, in this work we investigated (i) the effects of an ACC deaminase-producing endophytic bacterium on three different rhizobium-legume symbioses (one grain and two forage legumes) under the presence and absence of a high Mn concentration, and (ii) the role of an exogenous ACC deaminase in these rhizobial–legume symbioses. For that, the *Pseudomonas* sp. Q1 was selected from our lab collection of endophytic bacteria isolated from legumes [18,33], and its *acdS* gene was deleted in order to provide evidence of the importance of the activity of an exogenous ACC deaminase in the rhizobia and legume performance under stressed and non-stressed conditions. 

## 2. Results

### 2.1. Analysis of the acdS Gene Sequence from Pseudomonas sp. Q1

Data mining of the *Pseudomonas* sp. Q1 genome (Assembly number ASM993597v1) reveal the existence of genes related to ACC deamination. The *acdS* gene detected in the *Pseudomonas* sp. Q1 genome has a sequence of 1017 bp (Accession number MN232215) that encodes a protein of 338-amino acid residues with a predicted molecular weight of 36.9 kDa.

**Figure 1:**
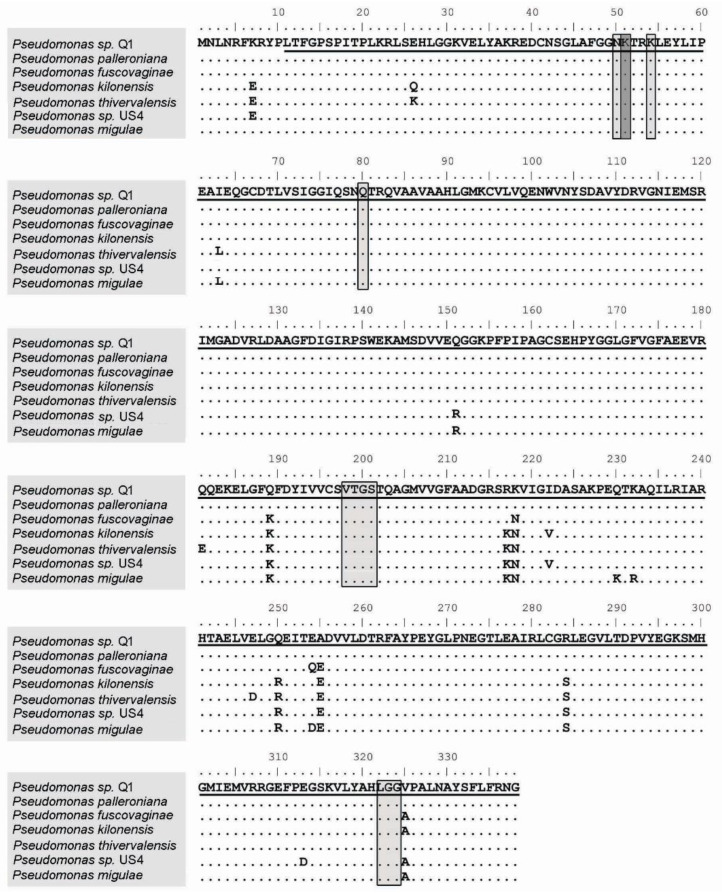
Alignment of AcdS protein amino-acid sequences from bacteria belonging to genus *Pseudomonas*. The conserved residues between all sequences are indicated by dots. The pyridoxal 5′-phosphate binding sites are indicated in light gray, and the catalytic residue is indicated in dark gray. The underlined amino acids correspond to the deletion fragment in *Pseudomonas* sp. Q1. *Pseudomonas palleroniana* accession WP_090372028, *Pseudomonas fuscovaginae* accession WP_019361456, *Pseudomonas kilonensis* WP_053178893, *Pseudomonas thivervalensis* accession WP_053183836, *Pseudomonas* sp. US4 accession WP_015096487.1 and *Pseudomonas migulae* accession WP_084321532.

To confirm the annotation of the putative *acdS* gene from *Pseudomonas* sp. Q1, an alignment of its predicted amino-acid sequence with the amino-acid sequence of several functional ACC deaminase proteins was performed (Figure 1). This alignment shows that the predicted *Pseudomonas* sp. Q1 ACC deaminase displays a high degree of similarity to the predicted and experimentally proven ACC deaminase enzymes from a variety of organisms, including several *Pseudomonas* species (Figure 1). The most similar protein is the ACC deaminase of *P. palleroniana* (100% identity, accession WP_090372028), followed by *P. fluorescens* (99.7%, accession WP_060756418), *P. fuscovaginae* (98.5%, accession WP_019361456), *P. kilonensis* (97.0%, accession WP_053178893), *P. thivervalensis* (96.8%, accession WP_053183836), and *P. migulae* (96.5%, accession WP_084321532). 

This alignment also showed that the *Pseudomonas* sp. Q1 *acdS* gene possesses both the pyridoxal 5′-phosphate binding sites and the catalytic residue (Figure 1) that are typical sites of the bacterial 1-aminocyclopropane-1-carboxylate deaminase (IPR005965) subfamily.

### 2.2. Confirmation of the ∆acdS Knockout Mutant and acdS-Complemented Strain Construction

The deletion of an internal fragment of 955 nt of the *acdS* gene sequence in the ∆*acdS* knockout mutant strain, as well as the introduction of the *acdS* gene plus its promoter and terminator regions (2200 nt) in the genome of the complemented strains were confirmed by PCR. Moreover, the detection of ACC deaminase activity in free-living conditions of *Pseudomonas* sp. Q1 but not its ∆*acdS* knockout mutant confirmed the presence of a functional *acdS* gene in this strain. The ACC deaminase activity of *Pseudomonas* sp. Q1 and the *acdS*-complemented strain was 3.78 and 3.32 µmol of α-ketobutyrate mg^−1^ protein·h^−1^, respectively. No ACC deaminase activity was detected either in the Δ*acdS* mutant strain or in any of the rhizobial strains used in this study. Furthermore, the acdS gene deletion in the ∆*acdS* knockout mutant strain did not affect its compatibility with any of the rhizobial strains in vitro. These results corroborate the fact that the ∆*acdS* knockout mutant phenotype is due to the loss of ACC deaminase functionality and not to other mutations that may occur during the mutant construction process.

### 2.3. Tolerance to a High Concentration of Mn

**Figure 2:**
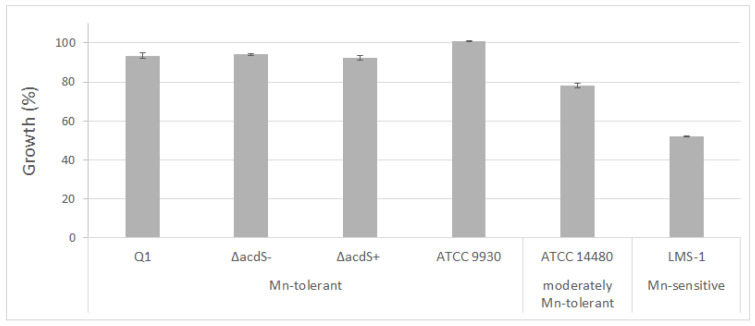
Growth of *Pseudomonas* sp. Q1 and its derivatives strains (∆*acdS* mutant and ∆*acdS*^+^) and rhizobial strains (*M. ciceri* LMS-1, *R. leguminosarum* ATCC 14480^T^, *E. meliloti* ATCC 9930^T^) under Mn conditions (2 mM MnSO_4_). Percentages were calculated considering the control condition (without addition of MnSO_4_) as 100% growth. Bars indicate standard deviation.

To evaluate Mn tolerance of the pseudomonads and rhizobial strains, their growth was evaluated in liquid medium under control (medium without additional Mn) and a high concentration of Mn. After 72 h, *E. meliloti* ATCC9930^T^ showed similar growth in the absence and presence of Mn. Similarly, when *R. leguminosarum* ATCC14480^T^ was exposed to a high level of Mn, its growth was only slightly slower than its growth under control conditions, indicating that this strain is moderately tolerant to Mn. On the other hand, the growth of *M. ciceri* LMS-1 was severely affected by Mn, as its growth was significantly lower in its presence compared to control conditions after 72 h (Figure 2). Altogether, *E. meliloti* and *R. leguminosarum* were considered to be Mn-tolerant, while *M. ciceri* was Mn-sensitive. 

The phenotype of the Δ*acdS* knockout mutant strain was also evaluated in the absence and presence of Mn compared to the wild-type strain. No significant differences between the growth rates of the Δ*acdS* knockout mutant and wild-type strain under those conditions were found (Figure 2). Moreover, *Pseudomonas* sp. Q1 and its derivatives showed similar growth characteristics both with and without Mn, indicating that *Pseudomonas* sp. Q1 is tolerant to these Mn concentrations and its ACC deaminase is not involved in its tolerance response to Mn.

### 2.4. Benefits of the Q1 Strain and of Its ACC Deaminase Activity on the Symbiotic Rhizobial-Legume Model 

From a preliminary assessment of the minimum concentration of Mn that constrains legume growth, it was determined that 30 mg L^−1^ of Mn is sufficient to significantly inhibit the growth of both chickpea and subterranean clover legumes (*data not shown*). The other two concentrations of Mn (45 and 60 mg L^−1^) applied to the plants greatly reduced the growth of the subterranean clover and were lethal to chickpea plants. For this reason, the concentration of 30 mg L^−1^ of Mn was chosen to assess the symbiotic performance of rhizobial strains under a high concentration of Mn and the effects of *Pseudomonas* sp. Q1 on the rhizobial-legume symbioses. 

#### 2.4.1. Chickpea-*Mesorhizobium* Symbiosis

**Figure 3:**
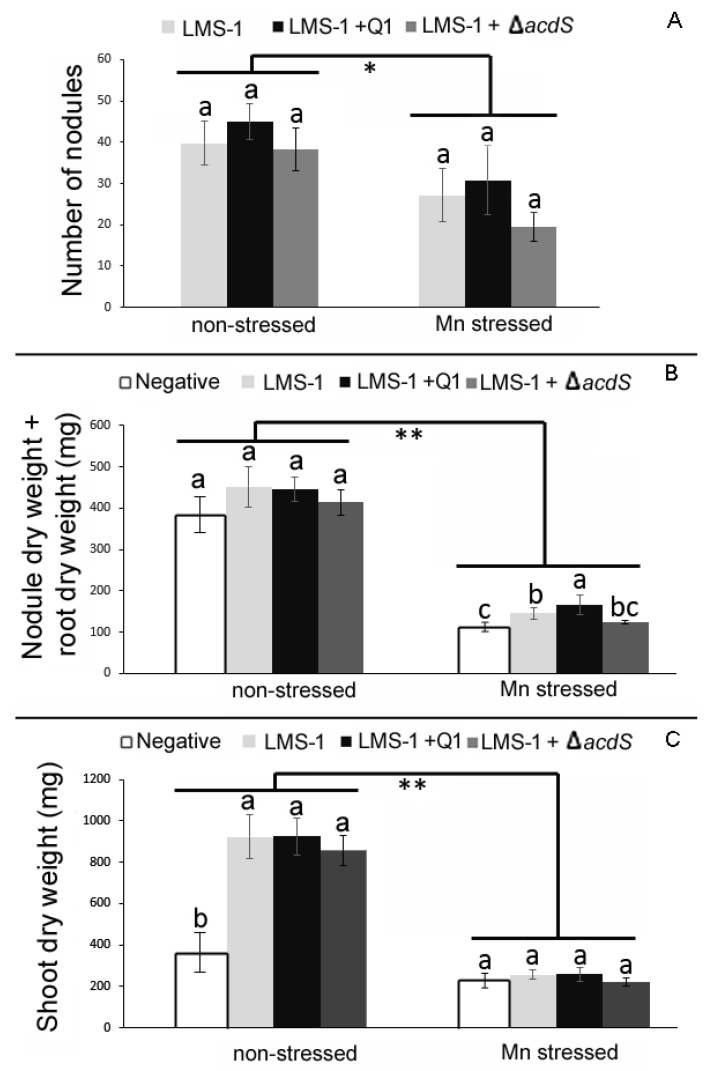
Effects of single inoculation (*M. ciceri* LMS-1 (LMS-1)) and co-inoculation (*M. ciceri* LMS-1 and *Pseudomonas* sp. Q1 (LMS-1 + Q1) or *M. ciceri* LMS-1 and *Pseudomonas* sp. Q1 ∆*acdS* mutant (LMS-1 + Δ*acdS*)) on chickpea plants grown in a vermiculite pot assay for 54 days. (**A**) Number of nodules of plants. (**B**) Nodule plus root dry weight of plants. (**C**) Shoot dry weight of plants. Each point represents the mean and standard error values of five plants per treatment. Different letters (a–c) correspond to statistically significant differences (*p* < 0.05) within each group (non-stressed and Mn stressed). * denotes statistically significant differences between the treatments means in the presence and absence of high Mn concentration (* *p* < 0.01; ** *p* < 0.001).

As previously observed [18], and compared to non-stressed conditions, the root and shoot dry weights of chickpea plants grown under a high concentration of Mn were significantly reduced either when single inoculated with the highly efficient strain *M. ciceri* LMS-1 [29,30,34] or co-inoculated with LMS-1 and Q1 strains (Figure 3). In fact, a statistically significant difference in average shoot and root dry weights by plant growth conditions was found (f(1) = 193.668, *p* < 0.001) for RDW and f(1) = 151.470, *p* < 0.001) for SDW). Furthermore, the chickpea plants co-inoculated with the LMS-1 and Q1 strains presented similar values in all parameters analyzed (number of nodules, total root and shoot dry weights) to the chickpea plants solely inoculated with LMS-1 strain in both non-stressed and Mn conditions. 

**Figure 4:**
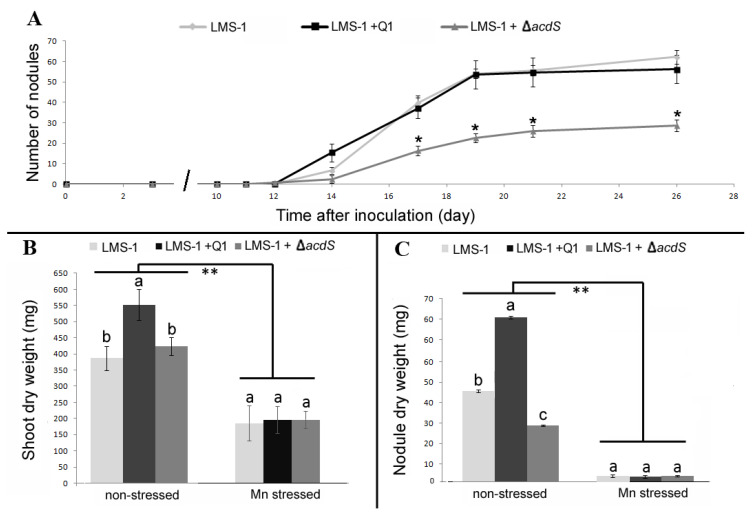
Nodulation kinetics by a hydroponic assay of chickpea plants single inoculated (*M. ciceri* LMS-1 (LMS-1)) and co-inoculated (*M. ciceri* LMS-1 and *Pseudomonas* sp. Q1 (LMS-1 + Q1) or *M. ciceri* LMS-1 and *Pseudomonas* sp. Q1 ∆*acdS* mutant (LMS-1 + Δ*acdS*)). (**A**) Nodulation kinetics of chickpea plants during 26 days at non-stressed conditions. Each point represents the mean and standard error values of eight plants per treatment. The asterisks correspond to significant differences between the LMS-1 + Q1 ∆*acdS* treatment and the other two treatments. (**B**) Shoot dry weight at the end of the hydroponic assay (**C**) Nodule dry weight at the end of the hydroponic assay. Different letters (a–c) correspond to statistically significant differences (*p* < 0.05) within each group (non-stressed and Mn stressed). * denotes statistically significant differences between the treatments means in the presence and absence of high Mn concentration (** *p* < 0.001).

In hydroponics, the nodulation kinetics showed that the co-inoculation of chickpea plants with LMS-1 and Q1 strains did not change the nodulation abilities (number of nodules and time of appearance) of the LMS-1 strain under non-stressed conditions (Figure 4A). However, plants co-inoculated with LMS-1 and Q1 strains formed bigger nodules, which is reflected in the significant increase of the nodule dry weight of those plants compared to plants single inoculated with LMS-1. Moreover, these bigger nodules may have contributed to the observed significant increase of the shoot dry weight of those plants compared to chickpea plants single inoculated with LMS-1 strain (Figure 4B). Under Mn conditions in hydroponics, the growth of chickpea plants was also severely affected, but no significant differences between treatments were observed for any of the plant parameters analyzed at 26 days after inoculation (DAI) (Figure 4C).

The hydroponic assay also revealed that the presence of the ∆*acdS* mutant strain in the chickpea—*M. ciceri* LMS-1 symbiosis contributed to a significant reduction of the number of nodules formed by the LMS-1 strain compared to plants inoculated with LMS-1 alone (Figure 4). As a result, the nodule and shoot dry weights in those plants were significantly lower than those obtained in plants inoculated with LMS-1 alone. Nevertheless, no significant differences were observed in any of the analyzed parameters of chickpea co-inoculated with the LMS-1 and ∆*acdS* mutant and chickpea single inoculated with LMS-1 in the pot assay, suggesting that the differences observed in hydroponics dissipate over time.

#### 2.4.2. Subterranean Clover-*Rhizobium* and Burr Medic-*Ensifer* Symbioses

**Figure 5:**
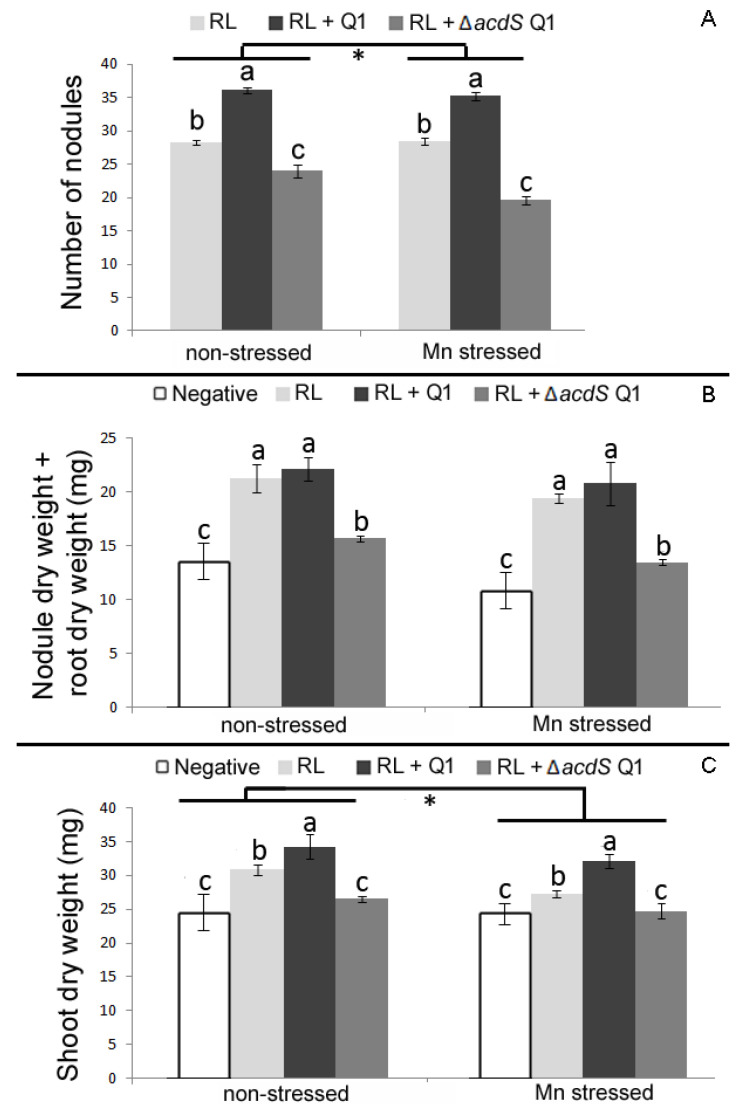
Effects of single inoculation (*R. leguminosarum* bv *trifolii* ATCC14480^T^ (RL)), and co-inoculation (*R. leguminosarum* bv *trifolii* ATCC14480^T^ and *Pseudomonas* sp. Q1 (RL + Q1) or *R. leguminosarum* bv *trifolii* ATCC14480^T^ and *Pseudomonas* sp. Q1 Δ*acdS* (RL + Δ*acdS* Q1)) on subterranean clover plants grown in a vermiculite pot assay for 42 days. (**A**) Number of nodules of plants. (**B**) Nodule plus root dry weight of plants. (**C**) Shoot dry weight of plants. Each point represents the mean and standard error values of five plants per treatment. Different letters (a–c) correspond to statistically significant differences (*p* < 0.05) within each group (non-stressed and Mn stressed). * denotes statistically significant differences between the treatments means in the presence and absence of high Mn concentration (* *p* < 0.01).

**Figure 6:**
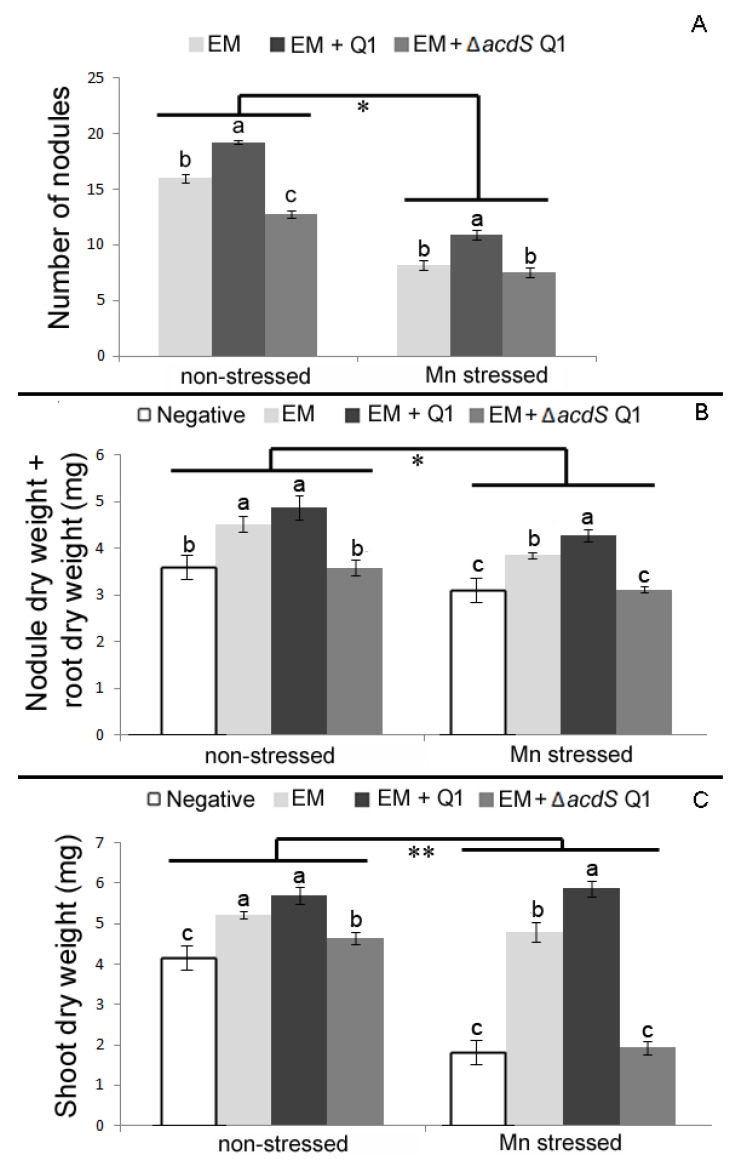
Effects of single inoculation (*E. meliloti* ATCC9930^T^ (EM)) and co-inoculation (*E. meliloti* ATCC9930^T^ and *Pseudomonas* sp. Q1 (EM + Q1) or *E. meliloti* ATCC9930^T^ and *Pseudomonas* sp. Q1 Δ*acdS* (EM + Δ*acdS* Q1) on burr medic plants grown in a vermiculite pot assay for 42 days. (**A**) Number of nodules of plants. (**B**) Nodule plus root dry weight of plants. (**C**) Shoot dry weight of plants. Each point represents the mean and standard error values of five plants per treatment. Different letters (a–c) correspond to statistically significant differences (*p* < 0.05) within each group (non-stressed and Mn stressed). * denotes statistically significant differences between the treatments means in the presence and absence of high Mn concentration (* *p* < 0.01; ** *p* < 0.001).

The growth of subterranean clover and burr medic species were also affected by Mn, although to a lesser extent than in chickpea plants. Compared to non-stressed conditions, the shoot dry weight of subterranean clover plants single inoculated with *R. leguminosarum* ATCC14480^T^ was significantly reduced (Figure 5), while the root dry weight of burr medic plants single inoculated with *E. meliloti* ATCC9930^T^ was significantly diminished under stressed conditions (Figure 6). 

The nodulation ability of *E. meliloti* was more impaired by Mn than it was for *R. leguminosarum* in plants inoculated with the rhizobia alone or with Q1 strain. The nodulation ability of the symbiotic strains *R. leguminosarum* and *E. meliloti* was significantly improved when their legume hosts (subterranean clover and burr medic) were co-inoculated with the non-rhizobial endophytic bacterium Q1 strain. This happens either under non-stressed or stressed conditions (Figure 5 and Figure 6). In both cases, the number of nodules formed by the microsymbionts was significantly enhanced by the presence of strain Q1 (Figure 5A and Figure 6A). Subterranean clover plants co-inoculated with *R. leguminosarum* and Q1 strains presented significantly higher values for shoot dry weight compared to plants inoculated with *R. leguminosarum* strain alone. In contrast, no significant differences were observed between the total root dry weights of single inoculated plants with rhizobium and plants co-inoculated with rhizobium and the Q1 strain. Interestingly, the positive effects of the Q1 strain on growth of both legume hosts were even more pronounced in plants exposed to high levels of Mn. For instance, the shoot dry weight values of both subterranean clover and burr medic plants were significantly higher in plants co-inoculated with their microsymbiont and the Q1 strain compared to plants inoculated with rhizobium alone under Mn toxicity. Moreover, these results are consistent with the significant increase in the number of nodules of those plants mentioned above.

**Figure 7:**
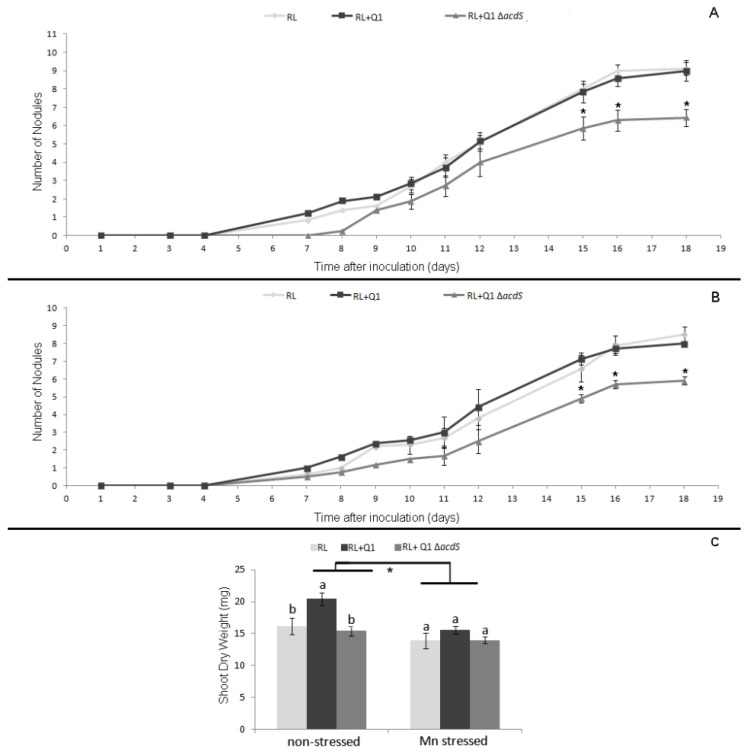
Nodulation kinetics by a hydroponic assay of subterranean clover plants inoculated with *R. leguminosarum* bv *trifolii* ATCC14480^T^ (RL), and co-inoculated with *R. leguminosarum* bv *trifolii* ATCC14480^T^ and *Pseudomonas* sp. Q1 (RL + Q1) or *R. leguminosarum* bv *trifolii* ATCC14480^T^ and *Pseudomonas* sp. Q1 Δ*acdS* (RL + Q1 Δ*acdS*). (**A**) Nodulation kinetics of subterranean clover during 18 days under non-stressed conditions. (**B**) Nodulation kinetics of plants during 18 days under Mn conditions. Each point represents the mean and standard error values of ten plants per treatment. The asterisks correspond to significant differences between the RL + ∆*acdS* mutant treatment and the other two treatments. (**C**) Shoot dry weight of subterranean clover at the end of the hydroponics experiments. Different letters (a–b) correspond to statistically significant differences (*p* < 0.05) within each group (non-stressed and Mn stressed). * denotes statistically significant differences between the treatments means in the presence and absence of high Mn concentration (*p* < 0.01).

During the first 18 days in hydroponics, no significant differences were observed in the nodulation kinetics of *R. leguminosarum* in subterranean clover single inoculated with this strain and plants co-inoculated with rhizobium and Q1 strains either under stressed or non-stressed conditions (Figure 7A,B). Nevertheless, the shoot dry weight of double inoculated plants was significantly higher compared to plants single inoculated with the *R. leguminosarum* strain at the end of the experiment under non-stressed conditions. On the other hand, no significant differences were observed between these treatments under stressed conditions. 

In contrast, both rhizobial–legume symbioses (subterranean clover*–R. leguminosarum* ATCC14480^T^ and burr medic*–E. meliloti* ATCC9930^T^) were negatively affected by the presence of the ∆*acdS* mutant strain. In fact, all parameters analyzed in the forage legumes co-inoculated with the ∆*acdS* mutant and the respective rhizobium were significantly reduced compared to plants inoculated solely with the respective microsymbiont (Figure 5 and Figure 6). Under stressed conditions, the presence of the ∆*acdS* mutant strain negatively affected the nodulation ability of *R. leguminosarum* (Figure 5). Nevertheless, the shoot dry weight of those co-inoculated plants were similar to those in subterranean clover plants inoculated solely with *R. leguminosarum*, suggesting that other plant growth-promoting mechanisms besides ACC deamination from strain Q1 may have helped the growth of subterranean clover in the presence of Mn. These results were consistent with the hydroponic data conducted with subterranean clover, where is observed that the nodulation kinetics of subterranean clover by *R. leguminosarum* were also impaired by the presence of the ∆*acdS* mutant either under stressed or non-stressed conditions (Figure 7A,B). Despite these differences, no differences between the shoot dry weight of plants double inoculated with its microsymbiont and the ∆*acdS* mutant and the plants inoculated with rhizobium alone were observed at the end of the hydroponic assay (Figure 7C). However, the nodulation ability of *E. meliloti* with burr medic was not affected by the presence of the ∆*acdS* mutant strain under Mn stressed conditions. Although the shoot dry weight and total root dry weight of plants co-inoculated with *E. meliloti* and ∆*acdS* mutant strains were significantly reduced compared to plants inoculated with *E. meliloti* alone (Figure 6).

## 3. Discussion

Mn toxicity is one of the most important constrains to plant growth on acid soils, especially for legumes dependent on N_2_-fixing symbiosis. Although the supplementation of acidic soils with dolomitic limestone alleviates the problem [10], it is an expensive approach and is not universally available. The introduction of highly efficient native rhizobia or engineered rhizobia strains can be useful tools as legume inoculants, particularly under challenging soil and climate conditions [35]. However, the selection and characterization of native rhizobial strains is a slow and expensive process, and the use of genetically modified organisms (GMO) is limited or prohibited in many countries. The present study evaluated the effects of the non-rhizobial endophytic bacterium *Pseudomonas* sp. Q1 on the symbiotic performance of three different rhizobia strains, as well as on the growth of their hosts in the absence and presence of high levels of Mn. 

As expected, all legume species were negatively affected by the presence of high levels of Mn, although expressing different levels of sensitivity. A previous study addressing the tolerance of pasture legumes to high concentrations of Mn in southern Australia revealed that in general, subterranean clover and related species are more tolerant to Mn than are *Medicago* species such as *M. polymorpha* [36]. Here, it was observed that the symbioses of these legume plants with their respective microsymbionts were differently affected by the presence of Mn. Even though *E. meliloti* is highly tolerant to Mn in free-living conditions, its ability to nodulate burr medic under stressed conditions was severely affected. On the other hand, no negative effect on the nodulation abilities of *R. leguminosarum* was observed under these Mn conditions, even though it displays lower tolerance to Mn under free-living conditions than *E. meliloti*. These data are consistent with the notion that the ability of legumes to grow in marginal soils is a consequence of the symbiotic associations that they are able to establish with nitrogen-fixing rhizobia [6,37,38]. Moreover, it has been reported that bacteria are capable of changing the chemical properties of rhizospheric soil and catalyzing redox reactions, altering heavy metal mobility and bioavailability, consequently reducing the toxicity of metals to plants [39,40,41,42]. In this context, the subterranean clover and burr medic symbioses with their respective microsymbionts, in the presence of Mn, may not only be a result of their nitrogen-fixation abilities but a cumulative result of several mechanisms employed by these microsymbionts; rhizobia possess a range of plant beneficial mechanisms other than nitrogen fixation [37,43]. 

Interestingly, the beneficial effects of the Q1 strain were only evident in the pasture legume–rhizobial symbioses while no effects were observed in chickpea plants inoculated with *M. ciceri* LMS-1. Despite these differences and the fact that *Pseudomonas* sp. Q1 was isolated from *Lupinus angustifolius* L., it can effectively colonize the internal root tissues of different plant species, including the legumes used herein (*Unpublished results*). The results presented here indicate that the presence of the endophytic non-rhizobial bacterial strain Q1 significantly increased the nodulation abilities of strains *R. leguminosarum* ATCC 14480^T^ and *E. meliloti* ATCC 9930^T^ in subterranean clover and burr medic, respectively, at the same time contributing to the growth promotion of these plants. The augmentation of legume nodulation through the co-inoculation of non-rhizobial bacteria along with rhizobia microsymbionts has been known for some time. For instance, the co-inoculation of a rhizobia and a *Bacillus* strain facilitated the nodulation of *Phaseolus acutifolius* by *Rhizobium* TAL 182, demonstrating the potential for root-associated non-rhizobial organisms to alter the dynamics of the legume–*Rhizobium* sp. symbiosis [44]. Similar results were reported with *Pisum sativum* [45], *Lotus* spp. [20], *Lens culinaris* [45,46], *Vigna radiata* [47], chickpea [48], and soybean [49,50] plants when co-inoculated with plant growth-promoting bacteria and with their rhizobia microsymbionts. 

Under Mn stress conditions, the beneficial effects of the Q1 strain on the symbiotic performance of ATCC 14480^T^ and ATCC 9930^T^ were even more evident. This is unexpected, since previous studies have shown that high concentrations of Mn and other metals can negatively affect ACC deaminase activity as well as other plant growth promoting properties [51,52]. High concentrations of Mn differently affected each of these rhizobium–legume symbioses. Nevertheless, in both cases, the presence of the Q1 strain facilitated the formation of nodules by these rhizobial strains and led to an increase of the host’s growth. These results are in agreement with previous reports showing that non-rhizobial bacteria stimulated legume growth and nodulation under different abiotic stress conditions, such as salinity [18,48,53,54,55,56,57], toxicity of copper [58,59,60], Mn [18], cadmium [23,61], and zinc [60]; as well as under biotic stress, namely root rot caused by *Fusarium solani* [56]. On the other hand, no beneficial effects of the co-inoculation of non-rhizobial bacterium along with *M. ciceri* LMS-1 were observed with the chickpea plants grown either under non-stressed or stressed conditions. Nevertheless, the lack of effects in the presence of the Q1 strain on the symbiotic performance of LMS-1 or chickpea growth under Mn toxicity conditions is most probably due to the high sensitivity of this symbiotic legume–rhizobium association to the Mn levels employed. 

The importance of ACC deaminase activity in plant–bacteria interactions, more specifically in the rhizobial nodulation process, has been demonstrated in several studies either by the transformation of rhizobial strains [28,29,30,31,62,63,64] or by combining rhizobia with ACC deaminase-expressing non-rhizobial bacteria [65,66,67,68,69,70,71]. Consistent with that, no beneficial effect of the Δ*acdS* mutant strain in the rhizobial–legumes symbioses was observed. This suggests that the ACC deaminase is a main plant growth-promoting mechanism responsible for the beneficial effects of the Q1 strain in the symbiotic subterranean clover–*R. leguminosarum* and burr medic–*E. meliloti* associations. In this regard, the presence of the ∆*acdS* mutant in co-inoculated plants resulted in a significant reduction of the rhizobial nodulation abilities and in the growth of the legume hosts either under non-stressed or stressed conditions. These results suggest that the importance of the ACC deaminase enzyme from *Pseudomonas* sp. Q1 in the rhizobium–legume symbiosis is not only on its ability to locally cleave the plant ethylene precursor ACC, which directly favors the nodulation process by the rhizobial strains, but additionally to prevent the activation of plant immune responses. Since ethylene is involved in plant immunity [72], ACC deaminase activity may block the activation of plant defenses in the roots [73]. For example, canola treated with ACC deaminase-producing *Pseudomonas putida* (formerly *Enterobacter cloacae*) UW4 down-regulated genes involved in ethylene-induced plant stress and in defense signaling pathways in comparison to canola plants inoculated with the ACC deaminase minus mutant of this bacterium [74]. Moreover, a transient induction of defense-related genes was observed in different legume species after rhizobial inoculation, and it was suggested that plant innate immunity is a crucial component in the establishment and maintenance of symbiosis [75]. 

Despite the fact that all rhizobial strains used in this study possess at least one copy of the *acdS* gene in their genomes, none of them presents the activity of this enzyme under free-living conditions. This may reflect the fact that rhizobial ACC deaminases contain a much lower level of this enzyme activity than do free-living soil bacteria [24]. Moreover, the expression of ACC deaminase in *Mesorhizobium loti* MAFF303099 and *M. ciceri* UPM-Ca7T occurs only inside of formed nodules [26,76] under the regulation of the NifA protein [77]. So far, this mechanism of ACC deaminase regulation has only been found in *Mesorhizobium* strains, suggesting that this regulatory mechanism is widespread in this genus [26,78]. It is possible that *M. ciceri* LMS-1 expresses ACC deaminase activity under symbiotic conditions, while the other two rhizobial strains used in this study do not. In this sense, it may be hypothesized that the role of ACC deaminase from strain Q1 in the tripartite associations containing *R. leguminosarum* ATCC 14480^T^ or *E. meliloti* ATCC 9930^T^ is important to overcome the plant defenses activated during the establishment of the rhizobium–legume symbiosis compared with the establishment of symbioses involving *M. ciceri* LMS-1, which could explain the less evident beneficial effects of the Q1 strain in this symbiotic association. In addition, the ACC deaminase may also affect other plant growth-promoting mechanisms that help to establish rhizobium–legume symbioses, especially those with low N_2_-fixing efficiency. A recent study revealed that *acdS* disruption in *Rahnella aquatilis* HX2 affected the regulation of other genes or enzymes related to plant growth-promoting features [79]. Thus, the ability of *R. aquatilis* HX2 to produce indole-3-acetic acid (IAA) and its antagonistic activity was diminished with the *acdS* disruption, while its nitrogen-fixing activity was increased. Similarly, the *acdS* disruption in strain Q1 may have affected the regulation of other plant growth-promoting mechanisms, altering the complementary of this strain with the rhizobial strains. Nevertheless, further studies on why the loss of ACC deaminase activity has caused deleterious effects on the pasture legume–rhizobial interactions are needed in order to better understand the precise nature of the benefits of exogenous ACC deaminase activity in the rhizobial–legume associations. 

## 4. Materials and Methods 

### 4.1. Bacterial Strains and Growth Conditions 

The bacterial strains and plasmids used in this study are listed in Table 1. The endophytic *Pseudomonas* sp. Q1, which was isolated from *L. angustifolius* L. roots and previously characterized [18], was selected to evaluate its effects on the symbiotic rhizobium–legume associations. Three rhizobial strains, namely *M. ciceri* LMS-1, *R. leguminosarum* bv. *trifolii* strain ATCC 14480^T^, and *E. meliloti* strain ATCC 9930^T^, were used as specific microsymbionts of chickpea, subterranean clover, and burr medic, respectively. The rhizobial strains were grown in yeast extract mannitol (YEM) medium [80] or in tryptone yeast (TY) medium [81] at 28 °C. Unless otherwise indicated, strain *Pseudomonas* sp. Q1 and its derivatives, Δ*acdS* knockout mutant and complemented strains, were grown in tryptic soy broth (TSB, Liofilchem) medium or tryptic soy agar (TSA, Liofilchem) medium at 28 °C, while the *Escherichia coli* strains were grown in Luria–Bertani (LB) *medium* [82] at 37 °C. Where indicated, 100 µg·mL^−1^ ampicillin, 25 µg·mL^−1^ chloramphenicol, or 10 µg·mL^−1^ tetracycline were added to the medium.

### 4.2. DNA Methods and Construction of Pseudomonas sp. Q1 Derivatives

#### 4.2.1. DNA Methods

The extraction of bacterial genomic DNA and plasmid DNA were conducted using the E.Z.N.A. bacterial DNA kit (Omega Bio-Tek, Norcross, GA, USA) and the Zyppy™ Plasmid Miniprep kit (Zymo Research, Freiburg, Germany), respectively, according to the manufacturer’s protocols. All PCR reactions were performed in a final volume of 50 μL containing 1× Buffer, 0.2 mM dNTPs, 1.5 mM MgCl_2,_ 15 pmol of each forward and reverse primers and 0.02 U/µL KOD Hot Start DNA Polymerase (Novagen, WI, USA). PCR products or gel bands were purified with the DNA Clean & Concentrator or Zymoclean^TM^ Gel DNA Recovery kits (Zymo Research, Freiburg, Germany). Unless otherwise specified, molecular techniques were performed using standard protocols [82]. The primers used in this study are listed in Table 2. 

#### 4.2.2. Construction of *Pseudomonas* sp. Q1 ∆acdS Mutant Strain

Based on the genome sequence of strain Q1 (SAMN09237650), the flanking regions of the *acdS* gene were amplified by PCR using primers designed as recommended for gene splicing by PCR-driven overlap extension [87]. The internal primers are designed to possess short complementary regions to each other (≈20 bp) that will hybridize with one another during the second PCR to serve as a chimeric template for the generation of a hybrid product [87]. PCR reactions to amplify the *acdS* flanking regions were performed using 10 ng of DNA and the following set of primers: acdS-Q1-Fa and acdS-Q1-Rd for upstream region and acdS-Q1-Fc and acdS-Q1-Rd primers for downstream region (Table 2). PCR reactions were performed using the followed programs: 95 °C for 2 min followed by 30 cycles of 95 °C for 20 s, 56 °C for 10 s (for upstream region) or 59 °C for 10 s (downstream region), and 70 °C for 21 s (upstream region) or 70 °C for 18 s (downstream region). 

To fuse the *acdS* flanking regions, another PCR reaction, containing 1 µL of a mixture of upstream and downstream-amplified products (±10 ng of each) as DNA template, was performed using the following program: 95 °C for 2 min, 30 cycles of 95 °C for 20 s, 56 °C for 10 s and 70 °C for 39 s. This reaction was performed using 15 pmol of the acdS-Q1-Fa and acdS-Q1-Rd primers (Table 2) and the DNA template (a mixture of upstream and downstream-amplified products) which possesses short complementary regions allowing the fusion between the upstream and downstream fragments, generating a hybrid product during the PCR amplification. 

The amplified product was purified, cloned in pNZY28-blunt vector (NZYtech, Lisbon, Portugal), and sequenced at StabVida Lda (Lisbon, Portugal). The fused-flanking region of the *acdS* gene was released from the pNZY28-blunt vector using the SmaI and XbaI sites and subcloned into the suicide plasmid pT18mobsacB, also linearized with the same restriction enzymes. Then, this product was transformed into *Escherichia coli* NZY5α competent cells (NZYtech, Lisbon, Portugal). Subsequently, these bacteria were used to mobilize the transformed pT18mobsacB into the *Pseudomonas* sp. Q1 strain by triparental mating, using the helper strain MT616 (pRK600) as described [29]. Double recombinants were selected as previously described [88].

#### 4.2.3. Complementation of *Pseudomonas* sp. Q1 ∆acdS mutant

##### pGRG36-Tet^r^ Vector Construction

A derivative of the pGRG36 vector with resistance to tetracycline was constructed and used to complement the Q1 ∆*acdS* mutant strain. To do this, the regulatory region of the *tetA* gene (tetracycline resistance gene) was amplified from the pT18mobsacB by PCR using the Tet-SdaI-Fw and Tet-SdaI-Rv primers (Table 2) and 20 ng of plasmid DNA. The amplification program was: 95 °C for 2 min followed by 30 cycles of 95 °C for 20 s, 65 °C for 10 s, and 70 °C for 53 s. The amplified product was cloned into pNZY28-blunt and then subcloned into the pGRG36 at SdaI restriction site to generate the pGRG36-Tet^r^. *Escherichia coli* DH5α transformed cells were selected on LB plates supplemented with tetracycline and glucose (0.1%) at 32 °C. 

##### Complementation 

To ensure that the ∆*acdS* knockout mutant phenotype is actually due to the loss of the functionality of the ACC deaminase, a complemented strain was obtained by restoring the ACC deaminase functionality in the ∆*acdS* knockout mutant strain. To complement the ∆*acdS* knockout mutant, the *acdS* gene and its regulatory regions were introduced into the chromosome of the mutant strain using the site-specific recombination machinery of the transposon *Tn7* present in pGRG36-Tet^r^. The putative regulatory regions of the *acdS* gene were identified using the software BPROM-Prediction of bacterial promoters [89] and ARNold Finding Terminators [90]. The DNA fragment carrying the complete *acdS* gene, its putative promoter and terminator regions were amplified using the primers acdS-Q1-Fa and comp_acds_Q1 Rv (Table 2) and 20 ng of plasmid DNA. The amplification program was 95 °C for 2 min followed by 30 cycles of 95 °C for 20 s, 57 °C for 10 s, and 70 °C for 55 s. The amplified product was purified and then cloned in pJET1.2/blunt vector (ThermoFisher Scientific, MA, USA). The extremities of the restricted BglII*-acdS* fragment were prepared using Fast end Repair Kit (ThermoFisher Scientific, MA, USA) to introduce the fragment into the pGRG36-Tet^r^ at its SmaI restriction site to generate the pGRG36-Tet^r^-*acdS*. The *E.coli* WM3064 transformed cells were selected on LB plates supplemented with tetracycline, 2,6-Diaminopimelic acid (DAP) (0.3 mM), and glucose (0.1%) at 32 °C, and they were used as a donor to introduce the pGRG36-Tet^r^ -*acdS* into the recipient the Q1 ∆*acdS* mutant strain by conjugation. After conjugation, selection of the ∆*acdS* mutant transformed cells was done by plating the mixture on TSA plates containing tetracycline and glucose (0.1%) at 23 °C for 48 h. To induce the expression of transposition genes in pGRG36-Tet^r^ -*acdS* and subsequent integration of DNA containing the *acdS* fragment into the chromosome of the Q1 ∆*acdS* mutant strain, colonies were grown at TSB supplemented with 0.1% arabinose at 23 °C on an orbital agitator. After 24 h, the bacterial cells were plated on TSA at 37 °C to cure the vector. The absence of pGRG36-Tet^r^ -*acdS in* ∆*acdS* cells and the integration of the *acdS* fragment into the chromosome of the ∆*acdS* mutant strain were confirmed by the inability of cells to grow in the presence of tetracycline and by PCR using the acdS-Q1-Fa and comp_acds_Q1 Rv, respectively.

### 4.3. ACC Deaminase Activity Assay 

The ACC deaminase activity was evaluated in strain Q1 and its derivatives, as well as in the rhizobial strains ATCC 14480^T^ and ATCC 9930^T^. *M. ciceri* LMS-1 was used as a negative control [29]. At 28 °C, *Pseudomonas* sp. Q1 and its derivatives were grown in TSB medium for 24 h, while the rhizobial strains were grown in TY medium for 2–3 days until they all reached stationary phase. Then, ACC deaminase activity was induced in these cells following the procedure described [29]. After induction, ACC deaminase activity was estimated based on the chemical determination of α-ketobutyrate that results from ACC cleavage by ACC deaminase [91], and the total protein contents of cells was quantified using the Bradford method [92] with bovine serum albumin as a standard. Final ACC deaminase activity was expressed in μmol α-ketobutyrate/mg protein/h.

### 4.4. Detection of acdS Genes by PCR

To detect the *acdS* gene in strains ATCC 14480^T^ and ATCC 9930^T^, two sets of primers were designed based on a conserved region of the *acdS* gene sequences from several *Rhizobium leguminosarum* bv. *trifolii* and *E. meliloti* strains. PCR reactions were performed using 20 ng of total DNA and the specific set of primers for each strain (Table 2). The amplification program was 95 °C for 2 min followed by 30 cycles of 95 °C for 20 s, 55 °C for 10 s, and 70 °C for 60 s. Sequencing reactions were performed by StabVida (Lisbon, Portugal). The sequences have been deposited in the GenBank database under the accession numbers: MN255490 and MN255491.

### 4.5. Compatibility between the Bacterial Endophyte Q1 or Its Derivatives and the Rhizobial Strains

To determine the compatibility between the endophytic bacterium Q1 or its derivatives and each of the rhizobial strains, a bioassay combining any strain of these two groups (a *Pseudomonas* and a rhizobial strain) was performed on YEM and TY plates to evaluate potential antagonist effects among them. At 28 °C, *Pseudomonas* sp. Q1 and its derivatives were grown in TSB medium for 24 h, whereas the rhizobial strains were grown in TY medium for 2–3 days on an incubator shaker at 200 rpm. Then, each bacterial culture was standardized to an initial OD_565 nm_ of 1.0. Each rhizobial cell suspension was spread uniformly on the entire plate and incubated for 2 h at 28 °C. Later, 10 µL of each endophyte Q1 or its derivatives culture were spotted on the previously prepared plates with rhizobial strains in triplicate. The plates were incubated at 28 °C and observed over a period of 96 h. Absence of a zone of inhibition between strains was considered as indicator of compatible strains. 

### 4.6. Evaluation of Strains’ Tolerance to High Concentration of Mn 

To evaluate Mn tolerance of the *Pseudomonas* sp. Q1, its derivative strains, and the rhizobial strains LMS-1, ATCC 14480^T^, and ATCC 9930^T^, the bacterial cells of each strain were grown in their respective liquid media supplemented or not with 30 mg·L^−1^ of Mn (MnSO_4_). Strain Q1 and its derivatives grew on M9 medium [24] while rhizobial strains grew on TY liquid medium. For each treatment, the bacterial culture was standardized to an initial OD_565 nm_ of 0.1 from an overnight growth. Then, the bacterial cultures were incubated in an orbital agitator at 28 °C for 24 h (strain Q1 and its derivative strains) or 72 h (rhizobial strains). After incubation, the bacterial suspensions were read by spectrophotometry at OD_565nm_. At least three replicas were performed per treatment under each condition. 

### 4.7. Effect of the Pseudomonas sp. Q1 and the ∆acdS Mutant Strains on the Rhizobia-Legume Symbioses

#### 4.7.1. Bacterial Inoculum Preparation 

The rhizobial strains, strain *Pseudomonas* sp. Q1, and the ∆*acdS* mutant strains were grown in TY medium or TSB medium, respectively, until they reached the late exponential phase at 28 °C. Then, the cells were washed twice and resuspended in sterile saline solution (0.85% *w/v* NaCl) or nitrogen-free nutrient solution [93] previously diluted 1:4 with sterile distilled water. The optical density of bacterial cultures at OD_565_ nm was recorded. For co-inoculation, the cell suspension of two bacterial isolates was mixed in a 1:1 ratio and vortexed to yield a homogenous suspension. 

#### 4.7.2. Seed Surface Sterilization and Germination

The chickpea (*Cicer arietinum* L. cv Elixir) seeds were surface-sterilized and pre-germinated as previously described [29]. The subterranean clover (*Trifolium subterraneum* L. cv Nungarin) and (*Medicago polymorpha* L. cv. Scimitar) seeds were surface sterilized by soaking in 70% (*v/v*) ethanol solution for 1 min, followed by immersion in a 0.1% (*w/v*) acidified mercuric chloride solution for 4 min. After disinfection, seeds were rinsed 10 times in sterile distilled water and incubated at room temperature for 2 h. Seeds were washed more three times with sterile distilled water and incubated overnight at 4 °C, with a subsequent incubation of 48 h at 28 °C. 

#### 4.7.3. Evaluation of the Minimum Concentration Levels of Mn with Significant Constraints for Legume Plants

In order to simulate the gnotobiotic assay conditions close to those found in soils of Southern Portugal in terms of Mn values [9], the minimum concentration of Mn that significantly constrains the growth of a grain (chickpea) and a forage (subterranean clover) legume was determined. A plant growth experiment was performed by testing five different Mn concentrations: no additional Mn (control), 15, 30, 45, and 60 mg·L^−1^ of Mn supplemented in the nutrient solution [94]. After the second week of legume growth, the nutrient solution was complemented with 140 mg·L^−1^ of nitrogen (KNO_3_) and with the respective additional Mn concentration. Five pots per treatment were used, and the nutrient solution was applied three times a week. After four weeks, plants were harvested and then dried, followed by the recorded of shoot dry weights. The minimum concentration of Mn causing significant damage to legume growth was considered as the lowest concentration of Mn in which the treatment showed a significant reduction in SDW in relation to that of plants without the addition of Mn (control).

#### 4.7.4. Pot Experiments under Gnotobiotic Conditions

To evaluate the effect of *Pseudomonas* sp. Q1 and the ∆*acdS* mutant strains on the symbiotic performance of three different rhizobial strains, plant growth assays were conducted in a growth chamber, under non-stressed and Mn stressed conditions using gnotobiotic systems. This system allows the study of individual biotic and abiotic factors that play important roles in plant–bacteria interactions in soil under controllable and reproducible conditions.

After seed germination, the seedlings were transferred to plastic pots filled with a sterile 1:1 (*v/v*) vermiculite:sand mixture. Chickpea seedlings (one per pot; ≈300 cm^3^) were immediately inoculated as earlier described by Nascimento et al. [29] while the other legume species seedlings (four per pot; ≈200 cm^3^) were inoculated only two days after sowing to ensure seedlings viability, with 250 µL of a bacterial suspension with an OD_565nm_ of 0.5 [95]. All pots were grown in a growth chamber with the light cycle, temperature, and relative humidity set up as described [34]. For non-stressed conditions, a nitrogen-free nutrient solution [94] was used to water the plants three times a week. For Mn-stressed conditions, Mn (MnSO_4_) was added to the nitrogen-free nutrient solution (at a final concentration of 30 mg L^−1^) after the second week of growth until the end of the experiment. Due to the sensibility of chickpea–*Mesorhizobium* symbiosis to Mn, chickpea plants were only subjected to Mn stress during the third week of the experimental assay. Three treatments for each legume-rhizobial combination were prepared as follows: (i) legume plants inoculated with a specific rhizobial strain alone; (ii) legume plants co-inoculated with a specific rhizobial strain and *Pseudomonas* sp. Q1; (iii) legume plants co-inoculated with a specific rhizobial strain and the Q1 ∆*acdS* mutant strain. Uninoculated plants were used as negative controls. Five pot replicates were used for each treatment. The chickpea plants were harvested 54 days after inoculation (DAI), while subterranean clover and burr medic were harvested at 42-DAI. For subterranean clover and burr medic, the Shoot and root dry weights were measured, and the number of nodules was recorded. 

#### 4.7.5. Hydroponic Assay

To evaluate the nodule kinetics and development, hydroponic plant-growth assays were conducted using *C. arietinum* and subterranean clover plants co-inoculated with either *Pseudomonas* sp. Q1 or the ∆*acdS* mutant strains, together with their specific microsymbiont. All procedures were based on the protocol described [34]. Bacterial suspensions with an OD_565 nm_ of 0.5 and 0.4 were used for inoculating *C. arietinum* and subterranean clover seedlings_,_ respectively. A nitrogen-free nutrient solution was used as a hydroponic solution [93]. When required, Mn (MnSO_4_) was added to the hydroponic solution at final concentration of 30 mg·L^−1^. Chickpea plants were grown for 26 DAI while subterranean clover plants grown for 18 DAI. The number of nodules was recorded during the experiment, while the shoot dry weights (for both plant species) and nodule dry weights (only for *C. arietinum* plants) were measured at the end of the experiment. Eight biological replicates were used for treatments with *C. arietinum* plants, while for treatments with subterranean clover plants, ten replicates were used. 

### 4.8. Statistical Analysis

Statistical analysis was carried out using IBM SPSS Statistics for Windows, version 21 (IBM Corp., Armonk, N.Y., USA). Data obtained from plant-growth assays were checked for normality and homogeneity of variances (Kolmogorov–Smirnov test and Levene’s test, respectively). One or two-way analysis of variance (ANOVA) were used to compare the treatments or groups means. The Duncan test was used to detect significant differences between the treatments or group means (*p* < 0.05). 

## 5. Conclusions

Overall, the data presented here indicate that dual inoculation of forage legumes with their respective microsymbiont and an ACC deaminase-producing non-rhizobial endophytic bacterium significantly improves the growth of these legumes even when they are growing under stressful levels of Mn. Moreover, these benefits are mainly due to the role of the ACC deaminase of the Q1 strain in favoring the establishment and maintenance of rhizobial–legume symbioses, probably by locally reducing the stress effects to the plants, or by overcoming the plant’s immunity defenses, or by regulating other complementary plant growth-promoting mechanisms that favor the tripartite interaction. Nevertheless, additional studies to identify the molecular mechanisms behind the benefits of these tripartite interactions would contribute to a better understanding of the complexity of the microbial interactions with legumes and thus facilitate optimizing the benefits of using beneficial bacteria in sustainable crop production. 

## Figures and Tables

**Table 1:** Bacterial strains and plasmids used in this study.

Plasmids or Bacteria	Description	Reference or Source
Plasmids		
pNZY28-blunt	Amp^r^, blunt ends	NZYtech
pJET1.2/blunt	Amp^r^, blunt ends	ThermoFisher Scientific
pT18mobsacB	plasmid suicide; Km^r^, sacB^s^	[83]
pRK600	Helper plasmid pRK2013, npt::Tn9, Cm^r^	[84]
pGRG36	Amp^r^, arabinose-inducible promoter, RP4 conjugal transfer origin, pSC101 temperature-sensitive origin, presenting *tnsABCD* genes for *Tn7* transposition	[85]
pGRG36-Tet^r^	pGRG36 derivative, Tet^r^	This work
pNZY28-∆*acdS*	regions flanking *acdS* in pNZY28	This work
pJET1.2-*acdS*	*acdS* gene and its promoter and terminator regions	This work
pT18mobsacB-∆*acdS*	regions flanking *acdS* in pT18mobsacB	This work
pGRG36-Tet^r^-*acdS*	*acdS* gene and its promoter region of *Pseudomonas* sp. Q1 strain in pGRG36‑Tet^r^	This work
*Pseudomonas* sp.		
Q1	*Pseudomonas* sp. Q1, *acdS*^+^	[18]
Q1 ∆*acdS*	Derivative of strain Q1, *acdS^-^*	This work
Q1 ∆*acdS^+^* complemented	Derivative of strain Q1 ∆*acdS*, *acdS*^+^ with a copy of *acdS* gene and its promoter and terminator region	This work
Rhizobia		
LMS-1	*M. ciceri*, *acdS*^+^, *nif*^+^, *nod*^+^, Nodulates chickpea (*Cicer arietinum*)	[26,34]
ATCC 14480^T^	*R. leguminosarum* bv. *trifolii*, Nodulates red clover *(Trifolium praetense)* and white clover *(Trifolium repens)*	ATCC^®^ Microbiome Standards
ATCC 9930^T^	*E. meliloti*, Nodulates *(Medicago sativa)* and sweet clover *(Melilotus alba)*	ATCC^®^ Microbiome Standards
*Escherichia coli*		
DH5α	Host for cloning	[82]
WM3064	Host for conjugation, presence of RP4 (*tra*) in the chromosome	[86]
MT616	Strain containing helper plasmid pRK600	[84]

Note: Ampicillin (Amp^r^), kanamycin (Km^r^), tetracycline (Tet^r^) and Chloramphenicol (Cm^r^) resistance. Sucrose sensitivity (SacB^S^). Presence (*acdS*^+^) or absence (*acdS*^-^) of *acdS* gene. Presence of nitrogen -fixation (*nif)*^+^ and *nodulation (nod*^+)^ genes.

**Table 2:** Primers used in this study.

Primer Names	Primer Sequence (5′→3′)	Application
Tet-SdaI-Fw Tet-SdaI-Rv	CCTGCAGGGGACAAGGGAAAACGCAAG CCTGCAGGCCGTCAGCGTTTTGTAATGG	*tetA* gene and its promoter and terminator regions (2624 bp)
acdS-Q1-Fa acdS-Q1-Rb	TCAAGAGGTTGACGGGTTCT CTGGACGCGAGCGTGAAGGGCGTGATGGGAGA	*acdS* upstream region (948bp)
acdS-Q1-Fc acdS-Q1-Rd	TCTCCCATCACGCCCTTCACGCTCGCGTCCAG AACATCGCAAAGACGTAGGG	*acdS* downstream region (898bp)
acdS-Q1-Fa comp_acds_Q1 Rv	TCAAGAGGTTGACGGGTTCT AATGCCCTCGATTGCCGGA	*acdS* gene and its promoter and terminator regions (1242 bp)
RL_acdS_all_Fw RL_acdS_all_Rv	AACGCTATCCGCTCACCTT ATCCCCTGCATCGACTTTC	*acdS* gene fragment (886 bp) from *R. leguminosarum* bv *trifolii* ATCC 14480^T^
EM_acdS_all_Fw EM_acdS_all_Rv	CAGCCGTCCCTGTAGTAATAGC GAAAAGTTCGAACGCTACCC	*acdS* gene fragment (1006 bp) from *Ensifer meliloti* ATCC 9930^T^
